# General practitioners’ stay-at-work practices in patients with musculoskeletal disorders: using Intervention Mapping to develop a training program

**DOI:** 10.1080/02813432.2023.2268674

**Published:** 2023-11-29

**Authors:** A. Møller, C. B. Bond, L. N. Andersen, J. Hartvigsen, M. J. Stochkendahl

**Affiliations:** a Research Unit for General Practice in Copenhagen, Department of Public Health, University of Copenhagen, Copenhagen, Denmark; b The Section of General Practice, Department of Public Health, University of Copenhagen, Copenhagen, Denmark; c Center for Muscle and Joint Health, Department of Sports Science and Clinical Biomechanics, University of Southern Denmark, Odense, Denmark; d Chiropractic Knowledge Hub, Odense, Denmark

**Keywords:** General practitioner, work participation, patient management, musculoskeletal disorders, sickness absence, Intervention Mapping, qualitative

## Abstract

**Objectives:**

To describe current stay-at-work practices among Danish general practitioners (GPs) in relation to patients with musculoskeletal disorders, to identify potential avenues for improvement, and to suggest a training program for the GPs.

**Design and Setting:**

We followed the principles of Intervention Mapping. Data were collected by means of literature searches, focus group interviews with GPs, and interaction with stakeholder representatives from the Danish labour market.

**Results:**

GPs’ current stay-at-work practices were influenced by systemic, organisational, and legislative factors, and by personal determinants, including knowledge and skills relating to stay-at-work principles and musculoskeletal disorders, recognition of the patient’s risk of long-term work disability, their role as a GP, and expectations of interactions with other stay-at-work stakeholders. GPs described themselves as important partners and responsible for the diagnostic and holistic assessments of the patient but placed themselves on the side line relying on the patient or workplace stakeholders to act. Their practices are influenced both by patients, employers, and by other stakeholders. We propose a training course for GPs that incorporate both concrete tools and behaviour change techniques.

**Conclusions:**

We have identified varied perspectives on the roles and responsibilities of GPs, as well as legislative and organisational barriers, and proposed a training program. Not all barriers identified can be addressed by a training course, and some questions are left unanswered, among others - who are best suited to help patients staying at work?

## KEY POINTS

1. Musculoskeletal disorders are highly prevalent and one of the most common causes for visiting a GP.
2. In many countries, GPs are important in facilitating that patients stay at work, when they are experiencing musculoskeletal pain and disability.
3. In our research, GPs place themselves on the side line as coaches relying on the patient or workplace to act.
4. Barriers such as role identity, systemic and organisational issues prevent GPs from being more involved in stay-at-work practices.
5. GPs’ with knowledge about stay-at-work practices may empower patients to better self-management.

## Introduction

In many countries, the general practitioner (GP) is an important person in encouraging and helping people stay at work despite living with chronic health problems and disability. Little is however known about how often work participation issues are discussed during regular GP consultations, what the content of the discussions is, or whether they affect work participation.

Musculoskeletal disorders are prevalent and one the most common causes for visiting a GP [1] as well as a common cause of sickness absence and early retirement [2]. Previous studies have identified that GPs can play an important role in helping people stay at work [3], but evidence also indicates that some patients with chronic musculoskeletal disorders become disillusioned, loose trust in the healthcare system [4], and expect nothing but sickness certification and/or pain medication from the clinical encounter with their GP [5].

In Denmark, GPs do not have to sanction sickness absence, which, since 2009, has been a matter of agreement between the employer and the employee. The GP may become involved, if the employer and employee fill in a ‘fit for work certificate’ (DK: *Mulighedserklæring*), which aims to clarify what the employee can do despite a disease or condition, and it is meant as a tool to keep the employee at work. The certificate is first filled in by the employer and employee, and then brought to the GP, who assesses whether the plan is feasible and adds suggestions to the plan if necessary. The ‘fit for work certificate’ was installed as one of several legislative initiatives to facilitate stakeholders to collaborate on finding the best work solution for the individual person. Despite the legislative initiatives, there are still barriers in the stay-at-work process. Therefore, we planned the development project (‘*Vejen Frem’*) [7] that focussed on identifying barriers and facilitators in the collaboration between GPs, patients with musculoskeletal disorders, and their employers about staying at work, and on developing interventions aiming at overcoming these barriers.

Due to changing demographics, the number of people living with age-related musculoskeletal disorders will increase, and since work ability decreases with age, stay-at-work practices must be improved [6]. Therefore, there is a need to understand how GPs can improve facilitation of work participation for people with musculoskeletal disorders. In this study, we used the Intervention Mapping framework (IM) to understand and describe current stay-at-work practices among Danish GPs in relation to patients with musculoskeletal disorders, to identify potential avenues for improvement, and to suggest a training program for the GPs.

## Materials and methods

### Overview

This study was conducted as part of a research and development project (‘*Vejen Frem’*) [7] that focussed on identifying barriers and facilitators in the collaboration between GPs, patients with musculoskeletal disorders, and their employers about staying at work, and on developing interventions aiming at overcoming these barriers. The project was conducted between August 2017 and April 2020 and followed the principles of IM [8]. IM is a systematic framework that leans on a combined use of both theory and empirical evidence for identifying needs for change and designing evidence-based interventions for complex health problems. IM is described in the literature as a series of six steps [8]; however, in reality, the designing (or planning) process is iterative and cumulative. The six steps are completed by means of active participation of stakeholders, and with a focus on individual-level, interpersonal-level, and environmental factors of health.

**Figure 1.**
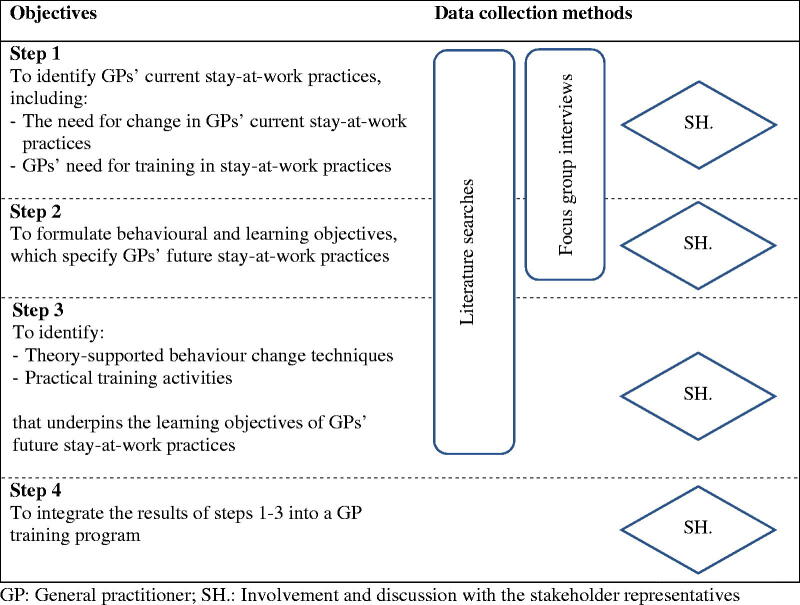
Objectives of Intervention Mapping steps 1–4 and the data collection methods. GP: General practitioner; SH.: Involvement and discussion with the stakeholder representatives.

In this project, which was a development project, we followed steps 1 to 4 of IM. Data were collected by means of three strategies: 1) several searches of scientific literature for existing theories and empirical evidence, 2) focus group interviews with GPs, and 3) workshops, meetings, and written communication with stakeholders. Throughout the duration of the project, data were collected iteratively so that information from the literature, interviews, and stakeholders mutually informed the next steps. The specific objectives of each IM step are presented in Figure 1 alongside the method of data collection related to each step. Details about the three data collection strategies are presented below.

### Literature searches

We systematically searched MEDLINE to identify qualitative, quantitative, and mixed-methods papers published in English, Danish, Norwegian, or Swedish using four blocks of search topics and derivatives with the Bolean operators AND and OR: 1) stay-at-work, 2) musculoskeletal pain, 3) general practitioners and 4) sickness certification. To reflect contemporary contexts and settings, we narrowed the search to the last ten years (January 2009 to April 2019). We prioritised systematic reviews, meta-analyses, and meta-syntheses, but supplemented these with primary literature when no reviews were available or more recent studies were identified. We also used feed-forward and citation tracking to identify additional papers as well as literature from the authors personal libraries. Finally, we searched MEDLINE and personal libraries for relevant behavior change theories and techniques to support the GP training program Results from the searches were screened for relevance to the topic, and eligible literature narratively described.

### Focus group interviews

We contacted well-established, general practice network groups in our personal networks in the Regions of Zealand and Southern Denmark and invited GPs to share their experiences on stay-at-work practices during focus group interviews. Further, an invitation was disseminated in Facebook groups for GPs in region Zealand. Three groups with 10 to 12 GPs from two different municipalities in Region Zealand and one in Southern Denmark agreed to participate, and on the days of the interviews, two, three and five GPs (five males and five females) showed up. All GPs worked in practices with multiple other GPs, had between five and 29 years of clinical experience in primary care and indicated that they discussed staying at work with patients between one and 15 times per week.

For the focus group interviews, we created a case vignette about a patient with musculoskeletal disorders who struggles to stay at work (supplementary file A) accompanied by an interview guide with prompts developed based on the literature searches and the research team’s experience as researchers and clinicians (supplementary file B). The interviews were conducted by MJS at locations convenient for the participants, audio- and video-recorded, and transcribed verbatim. Participating GPs were asked to draw on examples of cases from their own experience, and to bring forward additional topics not queried in the interview guides.

The interviews were then analysed following the systematic text condensation method by Malterud [9], which is a descriptive and explorative method for thematic analysis of qualitative data. The analysis followed four phases: 1) total impression from chaos to themes, 2) identifying and sorting meaning units from themes to codes, 3) condensations, from code to meaning and, 4) synthesizing from condensation to description and concepts [9]. In phase 1, authors MJS and AM individually read the transcripts and proposed preliminary themes and then met to discuss these. In phase 2, they identified meaning units in the data and sorted the data in codes according to the themes from phase 1 and new themes that emerged from their discussions. AM led the condensations in phase 3, and the final synthesizing in phase 4 was done in a collaboration between AM and MJS. Finally, condensates and quotations from each code group was extracted to produce an analytical text.

### Stakeholder involvement

A stakeholder group of 12 representatives from the Danish healthcare system and labour market contributed to’ *Vejen frem’* by discussing and prioritizing study findings from steps 1 and 2 (see Figure 1) and the appropriateness, relevance, and impact of potential interventions on GPs’ behaviour and environmental factors. The group included a GP, an occupational physician, a social case worker, a HR representative, and four worker and four manager representatives [7]. Three workshops with all stakeholder representatives, and a series of meetings with 1–3 stakeholders at the time, were held. Data from the workshops and meetings were collected as post-its, posters, and field notes. In addition, written comments on work documents were received from the stakeholders throughout the process.

### Ethical considerations

Data collection, storage and handling of personal data was approved by the Research and Innovation Office at the University of Southern Denmark under agreement with the Danish Data Protection Agency project number 18/3690 and submission number 10.689 and in accordance with the European General Data Protection Regulation. The project (file number 17/34518) was deemed exempt from ethical approval by the Regional Ethics Committee of Southern Denmark under the Danish legislation. Participation in the study was voluntary for all GPs, and written consent was obtained from all participants before the interviews.

## Results

Each step (step 1–4) in the IM process is presented below see also Figure 1 for an overview.

### Step 1: Understanding GPs current stay-at-work practices and need for change

Results from the literature search, focus groups interviews, and the stakeholder group’s discussions are presented separately. We then present a logic model of the problem based on the combined results to illustrate the potential need for change in GP practices.

#### Literature search

The literature search yielded 111 papers, of which three were included [10–12]. Forward citation tracking and searches of personal libraries yielded an additional 11 papers. The systematic search and resulting papers are presented in supplementary file C. Below, the findings from the included papers are presented as five topics.

##### Topic 1. Systemic reasons for not addressing work issues

One of the immediate obstacles presented in the literature was ‘GPs not addressing work issues or posing work-related questions’ [3,11,13]. Several reasons for this have been identified with the most frequently reported being lack of time during the clinical encounter [3,11,14]. Other environmental factors that impede addressing work participation are lack of financial incentives and standard procedures [3,14], and low status of occupational and social medicine among GPs [14].

##### Topic 2. GPs attitudes: Not within scope of practice

Not all GPs considered their patients’ work participation within their scope of practice or primary role [3]. Further, some expressed lack of confidence in doing functional capacity assessments [15,16]. Several studies pointed towards GP’s lack of knowledge of the compensation system [17] or their patients’ work tasks [10,18], resulting in GPs offering generic advice to patients rather than individualised, work specific advice [19]. Further, lack of knowledge of musculoskeletal disorders, biomedical orientations, GPs’ own fear-avoidance beliefs, and lack of compliance with work participation guidelines were identified as obstacles [3,12].

##### Topic 3. General practitioners’ actions depend on the patient’s complaints

GPs’ advice and willingness to certify sickness absence were strongly dependent on the somatic, psychological, and functional nature of the patients’ complaints [13], the perceived legitimacy of the complaints [15,20], and demands from the patient [21]. Patients presenting without a clear medical diagnosis, low severity of the complaint [13,21], and symptoms of pain, fatigue, and burnout [17], were less likely to be offered advice about staying at work [13].

##### Topic 4. Role conflict

Role conflicts faced by the GPs are important obstacles to work-focused clinical encounters. GPs are on one side the patient’s advocate and, on the other side, gatekeepers to statutory sickness absence benefits (in most countries) [3,11,20,21]. The role conflict has especially been identified in adversarial encounters with patients, where disagreement about indications for certifying sick leave [22], duration of sick leave, or functional assessment were aired [3]. Thus, GPs sometimes issued sick leave certificates even when they thought it was not strictly needed or legally required [15,20,23], and this role conflict was for some an obstacle for initiating work participation dialogues [11,13].

##### Topic 5. Communication and collaboration

Lack of communication and collaboration between GPs and relevant stakeholders have been identified as obstacles for patients’ work participation [3]. Specifically, lack of communication and coordination of work retention plans [3,16], one-way communication, and conflicting demands of the patient [14]. Drivers behind these were lack of common goals, trust [20], poor communicative skills, structural barriers, social norms, and not being used to involve others in their clinical practice [3]. Finally, lack of clear roles [17,23], responsibility, and standard procedures [14,23] have been reported as important barriers.

#### Focus group interviews

Based on our analysis, we found eight preliminary themes and identified seven code groups (Supplementary file D). Four of the seven code groups (the GPs’ biomedical perspectives; the patient’s life situation; the patient’s motivation for staying at work; and Timeout - a commonly used approach) were combined in the final analysis under the concept ‘It’s not all social medicine…’ - a biopsychosocial perspective, which describes aspects of the GPs’ actions and interactions with the patient. Data combined into the remaining two concepts led to identification of barriers to the GPs’ involvement in stay-at-work situations, namely the GPs’ self-perceived role and the collaboration with other stay-at-work stakeholders.

##### Concept 1. ‘it’s not all social medicine…’ - a biopsychosocial perspective

All GPs recognized the case in the vignette as a typical case. They described, how they were often approached by patients late in the process or as a final resort, which made them feel, it was too late for them to really make a difference. The GPs focused on a thorough clinical examination and posed a diagnosis, if possible. The aim of the examination was twofold; to establish the extent of the physical problem as a starting point for further actions, and to make the patient feel confident that the complaint was being taken seriously.

It cannot all be social medicine…there must be something physical, objective, we can take care of. (GP5)

After the initial assessment, GPs suggested a comprehensive psychosocial view on the patient’s life situation, ‘*it is the essence of being a GP*’ (GP5). The GPs described, how they took the patients’ resources and work-life balance into account, and how they experienced that psychological and social issues were important barriers for staying at work. In continuation, the patients’ individual motivation and personalities were perceived as important for staying at work.

But, it’s also the patient’s own storytelling. It becomes more and more rigid, and they start to tell a story about how ill they are, perhaps permanently ill. (GP7)

In addition to the clinical assessment and potential referral for further diagnostic testing or treatment, several GPs described an often used approach: ‘taking a timeout’. Here the patient called in sick for one or two weeks, and then used the time off work to consider his/hers options, and commonly, a second appointment to discuss the status after the timeout was scheduled (one or two weeks later). The GPs also described how they had experienced that the timeout could lead to prolonged sickness absence if the patient lacked motivation for returning to work, cancelled the appointment, or the GP forgot to follow up.

Some GPs had experienced that the request for a timeout came from the patient, and that sometimes the GPs questioned its legitimacy.

I think about this; is musculoskeletal pain an opportunity to pull the timeout card in a life that has become unmanageable? Or is the pain a barrier for work? There is a real difference… (GP6)

##### Concept 2. The GP’s self-perceived role in stay-at-work

The GPs described different perceptions of their role in handling stay-at-work issues, but many did not consider it a primary role.

And I don’t know if I can contribute with much else, because I think things are very complex. Of course, GPs have a role, partly in assessment and treatment, but also in the attitudes you convey to patients. From my perspective, we are not the most important partner in this. (GP4)

Further, very few GPs had ever contacted an employer directly. They did not see this as an important task, and they did not prioritize this in their daily work. Most described their role as supportive or as coaches who offered patients help to self-management. Some expressed a lack of competences in social medicine, which in turn led to uncertainty and unclear distribution of roles and responsibilities.

##### Concept 3. Collaboration with stay-at-work stakeholders

Overall, the collaboration and communication with other stakeholders such as employers or municipality case workers were sparse. Interestingly, not all GPs even saw the employers as important stakeholders. One GP mentioned only three partners: ‘the patient, us, and the municipality*’ (GP7).*

The GPs expressed varying experiences with the fit for work certificate, and its perceived usefulness was highly linked to the quality of input from the employers.

The municipal system was generally perceived as a barrier for the patients’ work participation despite its legislatively well-defined role and explicit task of facilitating work participation. It was clear from the interviews that collaboration between the municipalities and the GPs varied in both quality and trust. The GPs described how they had observed patients entering the municipal system and becoming more vulnerable because of bureaucracy.

If we take the…weakest group [of patients], who may not necessarily have been the weakest to begin with, but often become weak, it is those who enter the municipal humdrum…they wear out… (GP5)

One GP labelled this *‘system stress*’ (GP9). They had seen vulnerable patients trying to navigate different types of job training initiatives within the municipality with frequent changes of case managers. Several GPs described that they had even warned patients to stay out of the municipal system to avoid adversarial effects from entering the system.

You [the patient] must know, if this ends with sickness absence for much more than so and so, you will enter the municipal system, where all you can do is go along and do whatever you are being told. Sometimes this affects the motivation for some [patients]. (GP5)

Only two GPs mentioned the possibility of taking part in round table conferences with the patient, employer, and municipality, where the GPs are reimbursed for their time. Barriers for not participating related mostly to logistics such as scheduling of meetings and travelling to them. If these barriers could be overcome, positive attitudes towards the meetings were expressed.

#### The stakeholder group

Based on the findings from the literature search and the focus groups, the stakeholders discussed the most important barriers for patients successfully staying at work and the needs for change in relation to GPs’ stay-at-work practices, (Table 1).

**Table 1.** Environmental and behavioural barriers in relation to general practitioners’ involvement in stay-at-work situations identified by the stakeholder group.

**The most important environmental barriers that influence GPs’ behaviours are:** GPs, patients, employers, and other stakeholders have conflicting perspectives on how to facilitate stay-at-work Unclear roles and responsibilities between stakeholders in facilitating stay-at-work Lack of coordination of stay-at-work initiatives Lack of time and financial incentives for GPs to get involved in stay-at-work situations outside of the clinical encounter
**The most important GPs’ behaviours that need to change** Diagnosis-based or strictly biomedical approaches to patient assessments Lack of, inexpedient, or inappropriate communication with patients, employers, or other stakeholders

#### Logic model of the problem and overall goal

**Figure 2.**
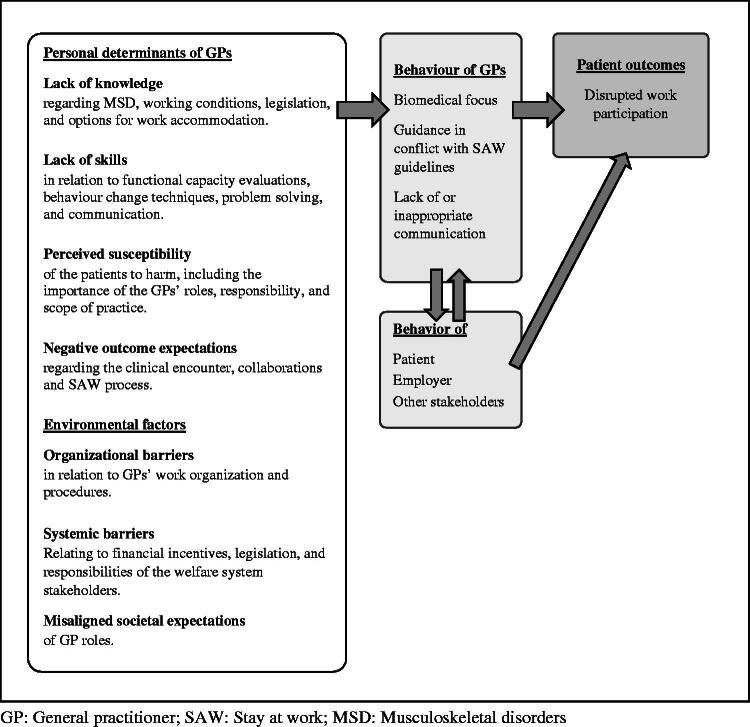
Logic model of the problem: General practitioners’ personal determinants and behaviours that impact their patients’ work participation. Based on results from step 1 in the Intervention Mapping framework (literature search, and focus group interviews). GP: General practitioner; SAW: Stay at work; MSD: Musculoskeletal disorders.

To visualise the combined results from step 1, we created a logic model of the problem as part of the IM framework (Figure 2).

The logic model illustrates how personal determinants and environmental factors may act as drivers of GP behaviour. The four most important personal determinants were: GPs’ lack of knowledge and skills [24], lack of recognition that the patient is at risk of long-term work disability (perceived susceptibility) [25], and GPs’ expectations of interacting with the patient and other stakeholders [26]. The model illustrates, how GP behaviour is influenced both by actions of and communication with patients, employers, and other stakeholders.

### Step 2: Formulating behavioural and learning objectives of GPs’ future stay-at-work practices

This step was informed by data from step 1, from both the literature search and the focus group interviews (Figure 1). Together with the stakeholder group, one objective relating to the GPs’ behaviour was formulated; *GPs act and communicate in a timely, collaborative, and problem-solving manner in the clinical encounter and in relation to the patient’s stay-at-work process*. Next, ten learning objectives (Table 2), specifying exactly what GPs need to do to fulfil the behavioural objective, were formulated, operationalised, and coupled. (Supplementary file E).

**Table 2.** Behavioural and learning objectives relating to general practitioners’ future stay-at-work practices.

**Behavioural objective:** GPs act and communicate in a timely, collaborative, and problem-solving manner in the clinical encounter and in relation to the patient’s stay-at-work process.
**Behavioural actions - the GPs will:** Act proactively with a focus on stay-at-work and early intervention Guide the patient in relation to self-management and stay-at-work Focus on functional capacity rather than a specific diagnosis Acquire relevant knowledge about the patient’s work, the working condition, and potential solutions (e.g. interventions offered from the compensation system) Assess the possibility for stay-at-work and potential need for sickness absence Advise the patient in concordance with guidelines for work participation Plan and suggest solutions both in the clinical encounter and in written communication with stakeholders Participate actively in communication with employers and other stakeholders Follow up on the patient’s progress Stay informed about updated work participation recommendations and guidelines

The most important barriers relating to GPs’ stay-at-work practices related to organizational and systemic factors, including lack of financial incentives and unclear formal role distribution among stay-at-work stakeholders. As changes in these barriers, would require legislative changes, the stakeholder group chose not to prioritize interventions targeting these barriers.

### Step 3: Identifying training activities

Based on the previous steps, relevant theory-supported behaviour change techniques were identified (Figure 1). The training activities were predominantly inspired by Bandura’s social cognitive theory [26], supplemented by a range of other social and learning theories [8,24,25]. In social cognitive theory, behavioural capability implies that if a person is to perform a particular type of behaviour, they must know the significance of the behaviour, its components (knowledge) as well as how to perform the behaviour (skills) [24,25]. Change methods in relation to knowledge included *discussion,* while the methods of *enactive mastery experiences* and *guided practice* were chosen to influence the determinant skills. Examples of learning objectives, behaviour change methods and practical applications are presented in Table 3, and a full overview is presented in Supplementary file F.

**Table 3.** Step 3: Examples of general practitioners’ learning objectives, and personal determinants for the general practitioners, behaviour change methods (and theory) and practical approaches.

Learning objectives and personal determinants	Behaviour change methods and (theories)	Practical approaches
Knowledge		
The GP has knowledge about: The positive impact of being proactive towards the patient’s work participation and negative consequences of delayed actions	***Discussion*** Encouraging consideration of a topic in open informal debate (Elaboration Likelihood Model)	GP engage in encouraging discussions with peer GPs about prevention, early proactive intervention, and the importance of helping the patient to self-manage, how to do it and when to do it
Skills		
The GP can: Apply behaviour change strategies directed at the patient and staying at work	***Guided practice*** Prompting individuals to rehearse and repeat the behaviour various times, discuss the experience, and provide feedback. (Social Cognitive Theory)	Using patient cases and under supervisor feedback, the GPs practice their abilities to identify situations where a proactive intervention would be beneficial and how to do it
	***Enactive mastery experiences*** Providing increasingly challenging tasks with feedback to serve as indicators of capability. (Social Cognitive Theory)	GPs are provided increasingly complex patient cases and given feedback by an expert to acquire relevant knowledge about the patient and find possible solutions
Perceived susceptibility		
GPs will recognize that: Patients with musculoskeletal disorders are at risk of having a recurrent or chronic condition and negative work-related consequences	***Consciousness raising*** Providing information, feedback or confrontation about causes, consequences, and alternative for a problem or a problem behaviour. (Health Belief Model)	GP are provided with up-to-date knowledge about musculoskeletal disorders and stay-at-work, including real-life cases, statistics, and expert comments
	***Environmental re-evaluation*** Encouraging the combination of cognitive and affective assessments of how the presence or absence of a personal behaviour affects one’s social environment. (Trans-Theoretical Model)	GPs are presented with patient cases to illustrate how the lack of a concrete plan increases the risk of a negative process for the patient
Outcome expectations		
The GP expects that: Early intervention will increase the patient’s likelihood of staying at work and decrease the likelihood of long-term sickness absence	***Environmental re-evaluation*** Encouraging realization of the negative impact of the unhealthy behavior and the positive impact of the healthful behavior. (Trans-Theoretical Model)	GPs are encouraged to discuss and share experiences on the impact of early interventions on the patient’s needs for sickness absence and work function
	***Shifting perspective*** Encouraging taking the perspective of the other. (Theories of Stigma and Discrimination)	GPs read and discuss patient stories to facilitate an understanding of how concrete plans increases the likelihood that they are implemented at the workplace

### Step 4: Integrating the results into a training program

We designed a two-day course targeting GPs that could be offered either as part of a larger national course or to local clusters of GPs. The course consists of an online module and physical attendance to upgrade knowledge, skills, perceptions, and outcome expectations. The content can be adapted to the GPs’ level of experience (i.e. pre- or post their medical specialization)

The course consists of four main elements:

1. Presentation of tools that the GP can use to aid the patient in staying at work, including screening of work-related barriers for stay-at-work [27], stay-at-work action points, problem solving [27,28], and goal setting.
2. Discussion of patient cases to a) increase GPs’ knowledge about musculoskeletal disorders and stay-at-work principles, b) gain insights into and increase the understanding of the impact of own actions, and c) increase the awareness and understanding of different stakeholders’ perspectives.
3. Pairwise and group-based discussions to facilitate knowledge sharing.
4. Practical assignments of increasing difficulty with peer and supervisor feedback, including screening for work-related barriers and problem solving with the patient, and performing and describing work role functioning including filling in the fit for work certificate.

During the course, the GPs will be asked to engage in short rounds of theory and hands-on assignments to facilitate practice and reflection of course content. The course should be held over two days approximately one month apart. Before the course, participants will be asked to reflect on their own practice and contribute with anonymized patient cases, including cases with a positive and negative outcome, and description of their own and the patient’s role in the case. Based on Tripp’s 4-step critical incident technique [29], the cases will be used to identify problems and potential solutions. Participants will then practice their newly acquired skills between course day 1 and 2 and reflect upon own practice.

In addition to the course, we suggest an online resource containing information and e-learning material made accessible to the GPs through the Danish healthcare system’s official online site *Doctor’s handbook* (‘Lægehåndbogen’), which is the primary resource for most GPs to search for information about diseases, conditions, procedures, and referrals. We suggest an update of the site to include entries on principles for staying at work, tools for dialogue with patients about staying at work, and assessment of functional capacity.

## Discussion

In this development project, we have aimed to understand current stay-at-work practice as a complex situation with the involvement of multiple stakeholders from different settings holding various viewpoints, and we used multiple data sources to increase the understanding. We consistently identified systemic, legislative, and organisational barriers for GPs being more involved in stay-at-work practices, including unclear roles and responsibilities between the GPs, patients, and workplace stakeholders. These factors appear to be significant, but they are not easily addressed without legislative changes. GPs described themselves as important partners and responsible for the diagnostic and holistic assessments of the patient, but at the same time, they placed themselves on the side line as coaches relying on the patient or on workplace stakeholders to take actions in relation to stay-at-work practices. If changes are to occur, several learning objectives must be met, including knowing more about the course and consequences of musculoskeletal disorders, knowing more about stay-at-work principles, being able to empower patients to actively self-manage their condition to stay-at-work, and knowledge and willingness among GPs to communicate with employers and other stakeholders.

Overall, the need for change identified from the Danish data sources mirrored the international literature on stay-at-work practices. The literature pertaining to stay-at-work is still relatively small compared to the return-to-work literature, but comparable topics about lack of stakeholder communication, collaboration, roles and responsibilities, and the GPs role conflicts emerged in both bodies of literature and data.

### Strengths and weaknesses of the study

It is a strength of the study, that we have used the IM framework to identify potential avenues for improvement, and to suggest a training program for the GPs. The framework has previously been used to facilitate continued work participation in various patient groups [30]. It has been shown to be particularly useful when conducting matrices of learning objectives and combining these objectives with theory-based approaches [31].

It is a strength, also according to the IM framework, that the proposed training program is based on specific learning objectives, behaviour change theories, and methods, and tailored to the GPs’ needs as identified in the needs assessment. Well-known pedagogical features have been added, such as an interval between course day 1 and 2 to allow time for reflection and contemplation and maintained behaviour change. Furthermore, the possibility for a partly online course may facilitate participation.

In relation to that, a limitation in this study is the challenges regarding the recruitment of GPs.

We have aimed for and invited a minimum of six GPs to each focus group; however, many participants did not show up for the scheduled interviews. From our previous experience with conducting primary health care studies, we know it is difficult to engage Danish GPs in research data collection, however, in this study; we encountered a more general lack of interest. This lack of interest and perceived relevance does not agree with the GPs’ relatively high weekly number of consultations concerning musculoskeletal problems that could include stay-at-work discussions, and the interview findings, where the GPs found the patient vignette clinically relevant. With the focus group interviews, we aimed to explore the field of interest, not to reach data saturation, and the data collection provided rich and in-detail descriptions of GPs’ current practices. Further, the systematic text condensation method enabled us to maintain a structure for interpreting data that led to reflections and thoughts of intersubjectivity [32]. Nonetheless, GPs who declined invitations or did not show up could have had other perspectives on stay-at-work practices than those presented here.

In this development project, we aimed for performing step 1–4 in the IM framework. It could be seen as a limitation to the study that we did not implement and evaluate the proposed training course for GPs. However, the project was only scheduled and funded for the first four steps.

### Generalisability and perspectives

The development project ‘Vejen frem’ has been performed in a Danish context. However, the literature search and the focus group interviews in step 1 showed that the stay-at-work practices were similar and met the same barriers internationally. Therefore, we assume that the results from the project can be used in other contexts, too. To fulfil the next steps in the IM framework, we are in discussion with the Danish GP post-graduate course organisers about adding the course to the post-graduate program. We suggest integrating future developments of training courses in the setting of the general practice quality development framework in clusters, which hopefully could lead to a higher degree of perceived relevance and importance for the GPs [33]. Further, formal evaluation of our training program is needed.

### Meaning of the study

In the future, GPs are expected to play even greater roles in all aspects of management of chronic diseases including helping patients stay at work for as long as they wish or need to [34]. We have identified various perspectives on the roles and responsibilities as well as barriers at systemic and organisational levels that cannot be addressed through GP training courses alone [35]. Ultimately, this leads to questions about who will be best suited to help the patients stay-at-work [36], and how they are best incentivised [35]. In the end, it matters more that patients receive the best possible help in maintaining a life with high social participation including participation in work life than *who* is involved in the management. In some countries, allied primary healthcare professionals like nurses [37], physiotherapists [38], chiropractors [39], occupational physicians and therapists, or social workers have successfully been assigned case manager roles. Regardless of how roles and responsibilities are distributed, the substantial national cost implications of discontinued work participation warrants improvements in the way our primary healthcare system and their actors incorporate and communicate about stay-at-work in their service for people with musculoskeletal disorders and other public health problems.

## Conclusions

In order to improve stay-at-work practices relating to patients with musculoskeletal disorders in general practice, we propose a training program that makes use of concrete tools and behaviour change techniques to target GPs’ knowledge, skills, recognition of the patients’ risk of long-term work disability and their expectations of interacting with the patient and other stakeholders. In addition, our analysis identified barriers that cannot be addressed by a training course alone, and some questions are left unanswered, among others - who are best suited to help patients staying at work?

## Supplementary Material

Supplemental Material
