# Supplementary material for: General practitioners’ stay-at-work practices in patients with musculoskeletal disorders: using Intervention Mapping to develop a training program
Source: Scand J Prim Health Care. 2023 Nov 29;41(4):445–56. doi: 10.1080/02813432.2023.2268674 (PMC11001345; doi:10.1080/02813432.2023.2268674)
Supplement: Supplemental Material [file IPRI_A_2268674_SM6631.docx]

**Supplementary files**

Supplementary file A: Case vignette used to start the focus group interviews with general practitioners

| Linda is 41 years of age, divorced and a single mom of two children aged 8 and 10 years.  She works as a cleaner on a local school. She has been absent from work multiple times due to back pain. She often has pain in her shoulder and arm while at work and at home.  She feels that her situation is no longer tenable but is in doubt what to do. She comes to you and asks if you can certify sick leave. |
| --- |

Supplementary file B: Example of an interview guide for the focus group interviews with general practitioners.

| **Construct** | **Questions** | **Prompts** |
| --- | --- | --- |
| **Opening questions** | Thinking about your patients, how do you recognise the situation described in the vignette? | - Do you see this type of patient in you daily work? - Can you give a recent example of similar/dissimilar situations? - How is the vignette similar/dissimilar to your experience? |
| **Patient characteristics** | In your experience, who would you identify as being challenged with staying at work? | - What would be a typical patient that you would most likely identify as having problems with staying at work? - What type of complaints/problems/specific characteristics does the patients have? – in relation to the physical complaint, life or work situation? |
| **Current practice** | If Linda was your patient, how you do handle the situation?  What would you advice a patient in a similar situation?  What do you think will happen to Linda/a patient in a similar situation? | - How do you think the outcome of the consultation will be? - What would you identify as important for a successful/unsuccessful clinical encounter with Linda/similar patient? Can you give examples? |
| **Interaction with other stakeholders** | Who would you identify as essential people that you have interacted with in Linda’s/similar situation? | - Who else might be involved in a similar situation? - What makes some people more essential? - How do you interact with [other stakeholders]? - What is the outcome of the interaction? |
| **Closing** | Is there anything you would like to add to the discussion?  Is there anything else that you think would be helpful for us to know |  |

Supplementary file C: Step 1. Systematic literature search and resulting papers.

**Search words**

| **Blok 1. Stay at work** | **Blok 2. Musculoskeletal pain** | **Blok 3. GPs** | **Blok 4. Sickness certification** |
| --- | --- | --- | --- |
| Employ*  Work  ”Work retention”  “Job retention”  ”Continue to work”  ”Stay at work”  “Sick leave”  ”Sickness absence”  ”Absenteeism”  ”Return to work”  “Worker’s compensation”  ”Sick benefits”  “Work disability” | Musculoskeletal pain  Musculoskeletal condition  Musculoskeletal dis*  Occupational dis*  Work-related dis*  Low back pain  Back pain  Neck pain | General practitioner  General practice  Family physician  Family practice  Primary care  Healthcare professional | Decision-making  Job accommodation  Accommodation  Disability management  Consultation  Sickness certification  Fit note |

**Search string and results**

Date of search: May 2, 2019.

Database: Ovid MEDLINE(R) and In-Process & Other Non-Indexed Citations and Daily 1946 to May 1, 2019 (kode: PMEZ)

| **#** | **Searches** | **Results** |
| --- | --- | --- |
|  |  |  |
| 1 | (Employ* or work or (work adj3 retention*) or (job adj3 retention*) or retention or (continu* adj3 work) or (stay* adj3 work) or sick leave or (sick* adj5 absen*) or absent* or (return adj2 work) or (worker* adj2 compensation) or (sick* adj4 benefit*) or work disability).ti,ab,kw. | 1621621 |
| 2 | (Musculoskeletal pain* or Musculoskeletal condition* or Musculoskeletal dis* or Occupational dis* or Work-related dis* or Low back pain* or Back pain* or Neck pain*).ti,ab,kw. | 73005 |
| 3 | ((General adj2 practi*) or (family adj2 phys*) or (family adj2 pract*) or (primary adj2 care) or (health?care adj2 profession*)). ti,ab,kw. | 224823 |
| 4 | (Decision?making or (Decision* adj3 mak*) or accommodation or (job adj3 accommoda*) or (work adj3 accommoda*) or (disability* adj3 manage*) or consult* or (sick* adj2 certificat*) or (fit adj2 note*)). ti,ab,kw. | 270308 |
| 5 | 1 and 2 and 3 and 4 | 223 |
| 6 | limit 5 to (yr="2009 -Current" and (danish or english)) | 111 |

**List of included and excluded papers**

|  | **First author** | **Titel** | **Source** | **Title and abstract screened** | **Full text screened** | **Included** |
| --- | --- | --- | --- | --- | --- | --- |
| 1 | Sturesson et al. | Quality in sickness certificates in a Swedish social security system perspective. | Scandinavian Journal of Public Health. 43(8):841-7, 2015 Dec. | x | x | x |
| 2 | Wynne-Jones et al. | Perceptions of health professionals towards the management of back pain in the context of work: a qualitative study. | BMC Musculoskeletal Disorders. 15:210, 2014 Jun 18. | x | x | x |
| 3 | Darlow et al. | The association between health care professional attitudes and beliefs and the attitudes and beliefs, clinical management, and outcomes of patients with low back pain: a systematic review. | European Journal of Pain. 16(1):3-17, 2012 Jan. | x | x | x |
| 4 | Nilsing et al. | Description of functioning in sickness certificates. | Scandinavian Journal of Public Health. 39(5):508-16, 2011 Jul. | x | x |  |
| 5 | Weevers et al. | Communication about work between general practitioners and patients consulting for musculoskeletal disorders. | Quality in Primary Care. 17(3):197-203, 2009. | x | x |  |
| 6 | Morris &  Watson | Investigating decisions to absent from work with low back pain: a study combining patient and GP factors. | European Journal of Pain. 15(3):278-85, 2011 Mar. | x | x |  |
| 7 | Hansen et al. | Retention in physically demanding jobs of individuals with low back pain: study protocol for a randomised controlled trial. | Trials [Electronic Resource]. 16:166, 2015 Apr 16. | x | x |  |
| 8 | Sanders et al. | The effectiveness of shared decision-making followed by positive reinforcement on physical disability in the long-term follow-up of patients with nonspecific low back pain in primary care: a clustered randomised controlled trial. | BMC Family Practice. 19(1):102, 2018 06 28. | x |  |  |
| 9 | Voigt-Radloff et al. | Well informed physician-patient communication in consultations on back pain - study protocol of the cluster randomized GAP trial. | BMC Family Practice. 20(1):33, 2019 Feb 25. | x |  |  |
| 10 | Stochkendahl et al. | Managing sickness absence of patients with musculoskeletal pain - a cross-sectional survey of Scandinavian chiropractors. | Chiropractic & manual therapies. 27:1, 2019. | x |  |  |
| 11 | Wynne-Jones et al. | Effectiveness and costs of a vocational advice service to improve work outcomes in patients with musculoskeletal pain in primary care: a cluster randomised trial (SWAP trial ISRCTN 52269669). | Pain. 159(1):128-138, 2018 01. | x |  |  |
| 12 | Reho et al. | Comparing occasional and persistent frequent attenders in occupational health primary care - a longitudinal study. | BMC Public Health. 18(1):1291, 2018 Nov 26. | x |  |  |
| 13 | Muralidhar et al. | Basic occupational health services (BOHS) in community primary care: the MSF (Dhaka) model. | BMJ Case Reports. 2017, 2017 Mar 20. | x |  |  |
| 14 | Sanders et al. | Acceptability of a vocational advice service for patients consulting in primary care with musculoskeletal pain: A qualitative exploration of the experiences of general practitioners, vocational advisers and patients. | Scandinavian Journal of Public Health. 47(1):78-85, 2019 Feb. | x |  |  |
| 15 | Gjesdal et al. | New episodes of musculoskeletal conditions among employed people in Norway, sickness certification and return to work: a multiregister-based cohort study from primary care. | BMJ Open. 8(3):e017543, 2018 03 14. | x |  |  |
| 16 | Nordeman et al. | Predictors for future activity limitation in women with chronic low back pain consulting primary care: a 2-year prospective longitudinal cohort study. | BMJ Open. 7(6):e013974, 2017 07 02. | x |  |  |
| 17 | Hyde et al. | Process and impact of patient involvement in a systematic review of shared decision making in primary care consultations. | Health Expectations. 20(2):298-308, 2017 04. | x |  |  |
| 18 | Oswald et al. | Work participation of patients with musculoskeletal disorders: is this addressed in physical therapy practice?. | Journal of Occupational Medicine & Toxicology. 12:27, 2017. | x |  |  |
| 19 | Ruseckaite et al. | Does medical certification of workers with injuries influence patterns of health service use?. | Work. 54(3):669-78, 2016 Jun 08. | x |  |  |
| 20 | Ruseckaite et al. | Factors associated with sickness certification of injured workers by General Practitioners in Victoria, Australia. | BMC Public Health. 16:298, 2016 Apr 06. | x |  |  |
| 21 | Rzewuska et al. | One-year trajectories of depression and anxiety symptoms in older patients presenting in general practice with musculoskeletal pain: A latent class growth analysis. | Journal of Psychosomatic Research. 79(3):195-201, 2015 Sep. | x |  |  |
| 22 | Lewis et al. | Should General Practitioners Issue a Sick Certificate to Employees Who Consult for Low Back Pain in Primary Care?. | Journal of Occupational Rehabilitation. 25(3):577-88, 2015 Sep. | x |  |  |
| 23 | Hubertsson et al. | Sick leave patterns in common musculoskeletal disorders--a study of doctor prescribed sick leave. | BMC Musculoskeletal Disorders. 15:176, 2014 May 24. | x |  |  |
| 24 | Hirsch et al. | Low back pain patient subgroups in primary care: pain characteristics, psychosocial determinants, and health care utilization. | Clinical Journal of Pain. 30(12):1023-32, 2014 Dec. | x |  |  |
| 25 | Reeuwijk et al. | How work impairments and reduced work ability are associated with health care use in workers with musculoskeletal disorders, cardiovascular disorders or mental disorders. | Journal of Occupational Rehabilitation. 24(4):631-9, 2014 Dec. | x |  |  |
| 26 | Nilsing et al. | Sickness certificates: what information do they provide about rehabilitation?. | Disability & Rehabilitation. 36(15):1299-304, 2014. | x |  |  |
| 27 | Nordeman et al. | Prognostic factors for work ability in women with chronic low back pain consulting primary health care: a 2-year prospective longitudinal cohort study. | Clinical Journal of Pain. 30(5):391-8, 2014 May. | x |  |  |
| 28 | Collie et al. | Sickness certification of workers compensation claimants by general practitioners in Victoria, 2003-2010. | Medical Journal of Australia. 199(7):480-3, 2013 Oct 07. | x |  |  |
| 29 | Roddy et al. | Musculoskeletal clinical assessment and treatment services at the primary-secondary care interface: an observational study. | British Journal of General Practice. 63(607):e141-8, 2013 Feb. | x |  |  |
| 30 | Jensen et al. | Do work-related factors affect care-seeking in general practice for back pain or upper extremity pain?. | International Archives of Occupational & Environmental Health. 86(7):799-808, 2013 Oct. | x |  |  |
| 31 | Georgy et al. | Met or matched expectations: what accounts for a successful back pain consultation in primary care?. | Health Expectations. 16(2):143-54, 2013 Jun. | x |  |  |
| 32 | Kristiansson et al. | Pain, power and patience--a narrative study of general practitioners' relations with chronic pain patients. | BMC Family Practice. 12:31, 2011 May 15. | x |  |  |
| 33 | Lotters et al. | Work and health, a blind spot in curative healthcare? A pilot study. | Journal of Occupational Rehabilitation. 21(3):304-12, 2011 Sep. | x |  |  |
| 34 | Cegolon et al. | The Primary Care Practitioner and the diagnosis of occupational diseases. | BMC Public Health. 10:405, 2010 Jul 09. | x |  |  |
| 35 | Norrmen et al. | The association of patient's family, leisure time, and work situation with sickness certification in primary care in Sweden. | Scandinavian Journal of Primary Health Care. 28(2):76-81, 2010 Jun. | x |  |  |
| 36 | Sallis et al. | Working towards a 'fit note': an experimental vignette survey of GPs. | British Journal of General Practice. 60(573):245-50, 2010 Apr. | x |  |  |
| 37 | Mallen & Peat | Discussing prognosis with older people with musculoskeletal pain: a cross-sectional study in general practice. | BMC Family Practice. 10:50, 2009 Jul 07. | x |  |  |
| 38 | Souville et al. | General practitioners and patients with psychological stress at work. | Journal of Occupational Rehabilitation. 19(3):256-63, 2009 Sep. | x |  |  |
| 39 | Lefevre-Colau et al. | Frequency and interrelations of risk factors for chronic low back pain in a primary care setting. | PLoS ONE [Electronic Resource]. 4(3):e4874, 2009. | x |  |  |
| 40 | Henschke et al. | Characteristics of patients with acute low back pain presenting to primary care in Australia. | Clinical Journal of Pain. 25(1):5-11, 2009 Jan. | x |  |  |
| 41 | Peterson et al. | Effectiveness of Models Used to Deliver Multimodal Care for Chronic Musculoskeletal Pain: a Rapid Evidence Review. | Journal of General Internal Medicine. 33(Suppl 1):71-81, 2018 May. | x |  |  |
| 42 | Adamsson et al. | Symptoms that may be stress-related and lead to exhaustion disorder: a retrospective medical chart review in Swedish primary care. | BMC Family Practice. 19(1):172, 2018 10 30. | x |  |  |
| 43 | Nuraydin A | The Mersin Greenhouse Workers Study. Surveillance of Work-related Skin, Respiratory, and Musculoskeletal Diseases. | Annals of Global Health. 84(3):504-511, 2018 Aug 31. | x |  |  |
| 44 | Bornhoft et al. | Health effects of direct triaging to physiotherapists in primary care for patients with musculoskeletal disorders: a pragmatic randomized controlled trial. | Therapeutic Advances in Musculoskeletal Disease. 11:1759720X19827504, 2019. | x |  |  |
| 45 | Seidman et al. | Collaborative care for a patient with complex low back pain and long-term tobacco use: a case report. | Journal of the Canadian Chiropractic Association. 59(3):216-25, 2015 Sep. | x |  |  |
| 46 | Michaleff et al. | Child and adolescent musculoskeletal pain (CAM-Pain) feasibility study: testing a method of identifying, recruiting and collecting data from children and adolescents who consult about a musculoskeletal condition in UK general practice. | BMJ Open. 8(6):e021116, 2018 Jun 14. | x |  |  |
| 47 | Riis et al. | Comparing satisfaction with a participatory driven web-application and a standard website for patients with low back pain: a study protocol for a randomised controlled trial (part of the ADVIN Back Trial). | Trials [Electronic Resource]. 19(1):399, 2018 Jul 25. | x |  |  |
| 48 | Zippelius et al. | Establishing an Interdisciplinary Compulsory Elective Subject 'Spine' in the Curriculum of the University Study Course of Human Medicine. | Acta Chirurgiae Orthopaedicae et Traumatologiae Cechoslovaca. 85(3):165-170, 2018. | x |  |  |
| 49 | Kim et al. | Characterizing Highly Frequent Users of a Large Canadian Urban Emergency Department. | The Western Journal of Emergency Medicine. 19(6):926-933, 2018 Nov. | x |  |  |
| 50 | Borgschulte et al. | Health care provision for refugees in Germany - one-year evaluation of an outpatient clinic in an urban emergency accommodation. | BMC Health Services Research. 18(1):488, 2018 06 25. | x |  |  |
| 51 | Healey et al. | The feasibility and acceptability of a physical activity intervention for older people with chronic musculoskeletal pain: The iPOPP pilot trial protocol. | Musculoskeletal Care. 16(1):118-132, 2018 03. | x |  |  |
| 52 | Bunzli et al. | Beliefs underlying pain-related fear and how they evolve: a qualitative investigation in people with chronic back pain and high pain-related fear. | BMJ Open. 5(10):e008847, 2015 Oct 19. | x |  |  |
| 53 | Hagen et al. | WITHDRAWN: Bed rest for acute low-back pain and sciatica. [Review] [33 refs] | Cochrane Database of Systematic Reviews. (6):CD001254, 2010 Jun 16 | x |  |  |
| 54 | Dahm et al. | Advice to rest in bed versus advice to stay active for acute low-back pain and sciatica. [Review] [29 refs] | Cochrane Database of Systematic Reviews. (6):CD007612, 2010 Jun 16 | x |  |  |
| 55 | Tan et al. | Predicting outcomes of acute low back pain patients in emergency department: A prospective observational cohort study. | Medicine. 97(26):e11247, 2018 Jun. | x |  |  |
| 56 | Bowman et al. | Multiple Practical Facts and Ideas to Improve Family Medicine Care. | Journal of the American Board of Family Medicine: JABFM. 30(6):687-690, 2017 Nov-Dec. | x |  |  |
| 57 | Darlow et al. | The Fear Reduction Exercised Early (FREE) approach to low back pain: study protocol for a randomised controlled trial. | Trials [Electronic Resource]. 18(1):484, 2017 Oct 17. | x |  |  |
| 58 | Walker et al. | Effect on healthcare utilization and costs of spinal manual therapy for acute low back pain in routine care: A propensity score matched cohort study. | PLoS ONE [Electronic Resource]. 12(5):e0177255, 2017. | x |  |  |
| 59 | Leutgeb et al. | Out of hours care in Germany - High utilization by adult patients with minor ailments?. | BMC Family Practice. 18(1):42, 2017 Mar 21. | x |  |  |
| 60 | Fritz et al. | Cost-Effectiveness of Primary Care Management With or Without Early Physical Therapy for Acute Low Back Pain: Economic Evaluation of a Randomized Clinical Trial. | Spine. 42(5):285-290, 2017 Mar. | x |  |  |
| 61 | He et al. | Depression influences pain and function after cervical disc arthroplasty. | Journal of Neurosurgical Sciences. 61(1):39-45, 2017 02. | x |  |  |
| 62 | Lingner et al. | Manual therapy applied by general practitioners for nonspecific low back pain: results of the ManRuck pilot-study. | Chiropractic & manual therapies. 26:39, 2018. | x |  |  |
| 63 | Vaucher et al. | The role of osteopathy in the Swiss primary health care system: a practice review. | BMJ Open. 8(8):e023770, 2018 Sep 01. | x |  |  |
| 64 | Hani & Liew | The views and experiences of Malaysian primary care doctors in managing patients with chronic low back pain: a qualitative study. | Malaysian Family Physician. 13(1):18-27, 2018. | x |  |  |
| 65 | Johnston | Consequences and management of neck pain by female office workers: results of a survey and clinical assessment. | Archives of Physiotherapy. 6:8, 2016. | x |  |  |
| 66 | Bishop et al. | A pilot cluster randomised controlled trial to investigate the addition of direct access to physiotherapy to usual GP-led primary care for adults with musculoskeletal pain: the STEMS pilot trial protocol (ISRCTN23378642). | Pilot & Feasibility Studies. 1:26, 2015. | x |  |  |
| 67 | Jensen et al. | Reproducibility of tender point examination in chronic low back pain patients as measured by intrarater and inter-rater reliability and agreement: a validation study. | BMJ Open. 3(2), 2013. | x |  |  |
| 68 | Al-Ahaideb et al. | Patterns and Obstacles of Provision of Minor Orthopedic Procedures among Primary Care Physicians in Saudi Arabia. | International Journal of Health Sciences. 6(1):13-21, 2012 Jan. | x |  |  |
| 69 | Skonnord et al. | Acupuncture for acute non-specific low back pain: a protocol for a randomised, controlled multicentre intervention study in general practice--the Acuback Study. | BMJ Open. 2(3), 2012. | x |  |  |
| 70 | Bolton & Hurst | Prognostic factors for short-term improvement in acute and persistent musculoskeletal pain consulters in primary care. | Chiropractic & manual therapies. 19(1):27, 2011 Nov 11. | x |  |  |
| 71 | Aho et al. | Patients referred from a multidisciplinary pain clinic to the social worker, their general health, pain condition, treatment and outcome. | Scandinavian Journal of Pain. 1(4):220-226, 2010 Oct 01. | x |  |  |
| 72 | Mansell et al. | Exploring What Factors Mediate Treatment Effect: Example of the STarT Back Study High-Risk Intervention. | Journal of Pain. 17(11):1237-1245, 2016 11. | x |  |  |
| 73 | Werner et al. | Cognitive Patient Education for Low Back Pain in Primary Care: A Cluster Randomized Controlled Trial and Cost-Effectiveness Analysis. | Spine. 41(6):455-62, 2016 Mar. | x |  |  |
| 74 | Schmidt et al. | Construct and predictive validity of the German Orebro questionnaire short form for psychosocial risk factor screening of patients with low back pain. | European Spine Journal. 25(1):325-32, 2016 Jan. | x |  |  |
| 75 | Lorenzo et al. | Acute low back pain management in primary care: a simulated patient approach. | Family Practice. 32(4):436-41, 2015 Aug. | x |  |  |
| 76 | Hill et al. | Development and Validation of the Keele Musculoskeletal Patient Reported Outcome Measure (MSK-PROM). | PLoS ONE [Electronic Resource]. 10(4):e0124557, 2015. | x |  |  |
| 77 | Conway et al. | Expanding access to rheumatology care: the rheumatology general practice toolbox. | Irish Medical Journal. 108(2):48-50, 2015 Feb. | x |  |  |
| 78 | Whitehurst et al. | Implementing stratified primary care management for low back pain: cost-utility analysis alongside a prospective, population-based, sequential comparison study. | Spine. 40(6):405-14, 2015 Mar 15. | x |  |  |
| 79 | Gehrt et al. | The role of illness perceptions in predicting outcome after acute whiplash trauma: a multicenter 12-month follow-up study. | Clinical Journal of Pain. 31(1):14-20, 2015 Jan. | x |  |  |
| 80 | Hider et al. | Pain location matters: the impact of leg pain on health care use, work disability and quality of life in patients with low back pain. | European Spine Journal. 24(3):444-51, 2015 Mar. | x |  |  |
| 81 | Foster et al. | Effect of stratified care for low back pain in family practice (IMPaCT Back): a prospective population-based sequential comparison. | Annals of Family Medicine. 12(2):102-11, 2014 Mar-Apr. | x |  |  |
| 82 | Aldrees et al. | Physician well-being: prevalence of burnout and associated risk factors in a tertiary hospital, Riyadh, Saudi Arabia. | Annals of Saudi Medicine. 33(5):451-6, 2013 Sep-Oct. | x |  |  |
| 83 | Frawley et al. | Prevalence and determinants of complementary and alternative medicine use during pregnancy: results from a nationally representative sample of Australian pregnant women. | Australian & New Zealand Journal of Obstetrics & Gynaecology. 53(4):347-52, 2013 Aug. | x |  |  |
| 84 | Mannion et al. | Association between beliefs and care-seeking behavior for low back pain. | Spine. 38(12):1016-25, 2013 May 20. | x |  |  |
| 85 | Leaver et al. | People seeking treatment for a new episode of neck pain typically have rapid improvement in symptoms: an observational study. | Journal of Physiotherapy. 59(1):31-7, 2013 Mar. | x |  |  |
| 86 | Beneciuk et al. | The STarT back screening tool and individual psychological measures: evaluation of prognostic capabilities for low back pain clinical outcomes in outpatient physical therapy settings. | Physical Therapy. 93(3):321-33, 2013 Mar. | x |  |  |
| 87 | Villaverde et al. | Motivations and objections to implement a spondyloarthritis integrated care pathway. A qualitative study with primary care physicians. | Reumatologia Clinica. 9(2):85-9, 2013 Mar-Apr. | x |  |  |
| 88 | Rodriguez et al. | Working with pain clinics and other consultants concerning low back pain. [Review] | Primary Care; Clinics in Office Practice. 39(3):547-52, 2012 Sep. | x |  |  |
| 89 | Del Pozo-Cruz et al. | A web-based intervention to improve and prevent low back pain among office workers: a randomized controlled trial. | Journal of Orthopaedic & Sports Physical Therapy. 42(10):831-41, 2012 Oct. | x |  |  |
| 90 | Mody & Brooks | Improving musculoskeletal health: global issues. | Best Practice & Research in Clinical Rheumatology. 26(2):237-49, 2012 Apr. | x |  |  |
| 91 | Fritz et al. | Primary care referral of patients with low back pain to physical therapy: impact on future health care utilization and costs. | Spine. 37(25):2114-21, 2012 Dec 01. | x |  |  |
| 92 | van Hooff et al. | A short, intensive cognitive behavioral pain management program reduces health-care use in patients with chronic low back pain: two-year follow-up results of a prospective cohort. | European Spine Journal. 21(7):1257-64, 2012 Jul. | x |  |  |
| 93 | Nordeman et al. | Prevalence and characteristics of widespread pain in female primary health care patients with chronic low back pain. | Clinical Journal of Pain. 28(1):65-72, 2012 Jan. | x |  |  |
| 94 | Paarup et al. | Prevalence and consequences of musculoskeletal symptoms in symphony orchestra musicians vary by gender: a cross-sectional study. | BMC Musculoskeletal Disorders. 12:223, 2011 Oct 07. | x |  |  |
| 95 | Muller-Schwefe | European survey of chronic pain patients: results for Germany. | Current Medical Research & Opinion. 27(11):2099-106, 2011 Nov. | x |  |  |
| 96 | Berenguera et al. | Study protocol of cost-effectiveness and cost-utility of a biopsychosocial multidisciplinary intervention in the evolution of non-specific sub-acute low back pain in the working population: cluster randomised trial. | BMC Musculoskeletal Disorders. 12:194, 2011 Aug 22. | x |  |  |
| 97 | Shiri &  Viikari-Juntura | Lateral and medial epicondylitis: role of occupational factors. [Review] | Best Practice & Research in Clinical Rheumatology. 25(1):43-57, 2011 Feb. | x |  |  |
| 98 | Hill et al. | Clinical outcomes among low back pain consulters with referred leg pain in primary care. | Spine. 36(25):2168-75, 2011 Dec 01. | x |  |  |
| 99 | Patel et al. | Study protocol: improving patient choice in treating low back pain (IMPACT-LBP): a randomised controlled trial of a decision support package for use in physical therapy. | BMC Musculoskeletal Disorders. 12:52, 2011 Feb 25. | x |  |  |
| 100 | Richard et al. | Self-efficacy and health locus of control: relationship to occupational disability among workers with back pain. | Journal of Occupational Rehabilitation. 21(3):421-30, 2011 Sep. | x |  |  |
| 101 | Mullis et al. | What does minimal important change mean to patients? Associations between individualized goal attainment scores and disability, general health status and global change in condition. | Journal of Evaluation in Clinical Practice. 17(2):244-50, 2011 Apr. | x |  |  |
| 102 | Grotle et al. | Are prognostic indicators for poor outcome different for acute and chronic low back pain consulters in primary care?. | Pain. 151(3):790-7, 2010 Dec. | x |  |  |
| 103 | Chaiter et al. | Quality control and quality assurance of medical committee performance in the Israel Defense Forces. | International Journal of Health Care Quality Assurance. 23(5):507-15, 2010. | x |  |  |
| 104 | Lonnberg et al. | Early predictors of the long-term outcome of low back pain--results of a 22-year prospective cohort study. | Family Practice. 27(6):609-14, 2010 Dec. | x |  |  |
| 105 | Hayden et al. | What is the prognosis of back pain?. | Best Practice & Research in Clinical Rheumatology. 24(2):167-79, 2010 Apr. | x |  |  |
| 106 | Rodriguez-Blanco et al. | Study protocol of effectiveness of a biopsychosocial multidisciplinary intervention in the evolution of non-specific sub-acute low back pain in the working population: cluster randomised trial. | BMC Health Services Research. 10:12, 2010 Jan 12. | x |  |  |
| 107 | Schmidt et al. | Assessing a risk tailored intervention to prevent disabling low back pain--protocol of a cluster randomized controlled trial. | BMC Musculoskeletal Disorders. 11:5, 2010 Jan 05. | x |  |  |
| 108 | Hill et al. | Subgrouping low back pain: a comparison of the STarT Back Tool with the Orebro Musculoskeletal Pain Screening Questionnaire. | European Journal of Pain. 14(1):83-9, 2010 Jan. | x |  |  |
| 109 | Lisi et al. | Characteristics of Veterans Health Administration chiropractors and chiropractic clinics. | Journal of Rehabilitation Research & Development. 46(8):997-1002, 2009. | x |  |  |
| 100 | Chesterton et al. | Transcutaneous electrical nerve stimulation for the management of tennis elbow: a pragmatic randomized controlled trial: the TATE trial (ISRCTN 87141084). | BMC Musculoskeletal Disorders. 10:156, 2009 Dec 11. | x |  |  |
| 111 | Thompson et al. | A sailor's pain: Veterans' musculoskeletal disorders, chronic pain, and disability. | Canadian Family Physician. 55(11):1085-8, 2009 Nov. | x |  |  |

Supplementary file D: Step 1. Focus group interviews. Overview of preliminary themes, codes groups and concepts

| **Preliminary themes identified** | **Code groups** | **Concepts** |
| --- | --- | --- |
| 1. A biomedical approach 2. The patient’s psychosocial situation 3. Patient reactions 4. Suggesting timeout | 1. General practitioners’ biomedical perspective 2. The patient’s life situation 3. The patient’s motivation for staying at work 4. Timeout - a commonly used approach | 1. “It’s not all social medicine…” - a biopsychosocial perspective |
| 1. Not our job 2. Not time for this | 1. Self-perceived role | 1. The general practitioners’ self-perceived role in stay-at-work situations |
| 1. The municipality 2. Fit for work certification | 1. The role of the municipality 2. Collaborating with employers | 1. Collaboration with stay-at-work stakeholders |

Supplementary file E. Step 3: Operationalised learning objectives related to the behavioural outcome: *General practitioners act and communicate in a timely, collaborative, and problem-solving manner in the clinical encounter and in relation to the patient’s stay-at-work (SAW) process.*

| **Learning objectives**  ***The GP will:*** | **Personal determinants** | | | |
| --- | --- | --- | --- | --- |
|  | **Knowledge** | **Skills** | **Perceived susceptibility** | **Outcome expectations** |
| (1) Act proactively with focus on SAW and early intervention | Describe positive impact of being proactive towards the patient and negative consequences of delayed actions  Describe proactive actions regarding SAW in both work and non-work MSD consultations  Recognize situation in which proactive actions could be useful | Demonstrate skills to identify situations where a proactive effort may positively impact on the patient and to initiate this effort | Recognize that patients with MSD are at risk of having a recurrent or chronic condition  Recognize that patients with MSD are at risk of negative work-related consequences | State that early intervention will increase the patient’s likelihood of SAW and decrease the likelihood of long-term sickness absence |
| (2) Guide the patient in relation to self-management and SAW | Describe principles of self-efficacy and self-management in relation to SAW  Recognize low self-efficacy and self-management in patients  Describe behaviour change strategies that can be used together with the worker to increase SAW | Express confidence in the ability to apply behaviour change strategies with the patient  Demonstrate ability to apply behavior change strategies directed at the patient and SAW | Recognize that patients who rely strongly on the GP are at risk for becoming passive and unable to manage their work-related situation  Recognize that patients with low levels of self-management are at risk for negative consequences of MSD and decreased likelihood of SAW | State that initiating a dialogue with the patient about self-management will increase the likelihood of SAW |
| (3) Focus on functional capacity rather than a specific diagnosis | Describe the principles of evaluations of work-related functional capacity | Demonstrate ability to evaluate functional capacity through patient history and objective assessment. | - | State that performing assessments of functional capacity will increase the patient’s likelihood of SAW by focusing on what the patient can do, rather than what the patient cannot do |
| (4) Acquire relevant knowledge about the patient’s work, working conditions, and potential SAW solutions (e.g. interventions offered by the compensation system) | List work-related risk factors that must be assessed in the patient case story  List available resources outside the GP’s domain and potential resource persons  Describe options for early SAW interventions | Demonstrate the ability to enquire about work-related factors in the patient case story | Recognize that the patient’s risk for negative consequences of MSD increase with their lack of knowledge about their SAW rights and responsibilities | State that discussing SAW options with the patient will increase the patient’s likelihood of getting an individually tailored solution |
| (5) Assess the possibility for SAW and need for sickness absence | List the positive impact of SAW and part-time sickness absence and negative impact of full-time sickness absence  Recognize situations, where the worker would benefit from part-time sickness absence rather than full-time | Demonstrate the ability to discuss the positive impact of SAW | Recognize that patients on full-time sickness absence are at risk of long-term sickness absence and work termination | State that discussing SAW with the patient will reduce the patient’s likelihood of negative, work-related consequences |
| (6) Advice the patient in concordance with guidelines for work participation | Describe current best practice of SAW  Recognize situations where these principles are difficult to apply, or the clinical encounter may be challenging  Describe strategies for SAW discussions during challenging clinical encounters | Express confidence in their ability to handle challenging clinical encounters in concordance with SAW principles  Demonstrate ability to discuss and advise about SAW during challenging clinical encounters | Recognize that the patient is in a difficult life situation and may have different expectations about SAW | Expect that advising according to guidelines will help the patient SAW |
| (7) Make a plan and suggest solutions both in the clinical encounter and in written communication with stakeholders | List potential SAW solutions (including early interventions and the fit-for-work certificate) | Demonstrate ability to transform general SAW solutions to individually tailored solutions  Demonstrate the ability to make a plan together with the patient and other stakeholders | Recognize that the patient’s likelihood of  SAW may decrease if the solution is not specific or tailored to the patient and the workplace  Recognize that the patient’s likelihood of  SAW may decrease if the solutions are not specifically planed and communicated with the patient and relevant stakeholders | State that specific solutions will increase the likelihood of workplace implementation |
| (8) Participate actively in communication with employers and other stakeholders | Describe the purpose of the fit for work certificate.  List content written by the GP that may help or hinder the patient’s SAW  Describe the purpose of round table meetings, and list strategies for prioritizing participation | Demonstrate ability to complete a fit for work certificate | Recognize that lack of collaboration with other stakeholders may increase the risk for uncoordinated guidance and solutions for the patient.  Recognize that the worker conveys messages from other stakeholders, and this entails the risk for information loss and misunderstanding | State that active collaboration between stakeholder will increase the patient’s likelihood of SAW |
| (9) Follow up on the patient’s progress | Describe the positive impact of follow-up on the patient’s progress and the potential negative impact of not following up  Recognize situations that require a change in plans | Demonstrate the ability to plan and carry through follow up consultations and revision of plans | - | State that the patient’s likelihood of SAW may increase with follow up consultations and adjustment of plans |
| (10) Stay informed about updated SAW recommendations and guidelines | List resources for staying updated  Describe their personal strategi for staying updated | - | Recognize that the patient’s likelihood of receiving out-dated advice increase with time | State that staying updated decrease the likelihood of the GPs being frustrated about the process |

MSD: Musculoskeletal disorders; GP: General practitioner; SAW: stay-at-work

Supplementary file F. Overview of learning objectives, behaviour change methods and theories, and practical approaches in the training program.

| Learning objectives and personal determinants | Behaviour change methods and theories (in brackets) | Practical approaches |
| --- | --- | --- |
| Knowledge |  |  |
| The GP has knowledge about:   - The positive impact of being proactive towards the patient and negative consequences of delayed actions - Proactive actions regarding SAW in both work and non-work MSD consultations - Situation in which proactive actions could be useful - Principles of self-efficacy and self-management in relation to SAW - Patients’ self-efficacy and self-management - Behaviour change strategies that can be used together with the worker to increase SAW - Principles of evaluations of work-related functional capacity - Work-related risk factors that must be assessed in the patient case story - Available resources outside the GP’s domain and potential resource persons - Options for early SAW interventions - The positive impact of SAW and part-time sickness absence and negative impact of full-time sickness absence - Situations, where the worker would benefit from part-time sickness absence rather than full-time - Current best practice of SAW - Situations where these principles are difficult to apply, or the clinical encounter may be challenging - Strategies for SAW discussions during challenging clinical encounters - Potential SAW solutions (including early interventions and the fit-for-work certificate) - The purpose of the fit for work certificate. - Content of the fit for work certificate written by the GP that may help or hinder the patient’s SAW - The purpose of round table meetings, and list strategies for prioritizing participation - The positive impact of follow-up on the patient’s progress and the potential negative impact of not following up - Situations that require a change in plans - Resources for staying updated - Their personal strategi for staying updated | ***Advance organizers***  Presenting an overview of the material that enables a learner to activate relevant schemas so that new material can be associated  (Theories of Information processing) | Easily assessable information material with easy-to-use overviews and guidance on MSD and SAW procedures |
|  | ***Discussion***  Encouraging consideration of a topic in open informal debate  (Elaboration Likelihood Model) | Encourage discussions with peer GP’s about:  - prevention and early proactive intervention  - the importance of helping the patient to self-manage, how to do it and when to do it  - principles for the assessment of functional capacity  - the benefits of part-time sickness absence  - SAW principles and solutions, when they can be used and when they are difficult to use.  - the use of fit for certificates, bit falls and strategies for communication with other stakeholders.  - the advantages and disadvantages of following-up and how to act based on the follow-up.  - strategies for staying updated on SAW recommendations and guidelines |
|  | ***Elaboration***  Stimulating the learner to add meaning to the information that is processed.  (Theories of Information Processing) | The GPs are encouraged to write down how they plan to communicate with other stakeholders when similar situations occur and their personal strategy for staying up to date |
| Skills |  |  |
| The GP has the ability to:   - Apply behaviour change strategies directed at the patient and SAW - Evaluate functional capacity through patient history and objective assessment. - Enquire about work-related factors in the patient case story - Discuss the positive impact of SAW - Discuss and advise about SAW during challenging clinical encounters - Transform general SAW solutions to individually tailored solutions - Make a plan together with the patient and other stakeholders - Complete a fit for work certificate - Plan and carry through follow up consultations and revision of plans | ***Guided practice***  Prompting individuals to rehearse and repeat the behavior various times, discuss the experience, and provide feedback.  (Social Cognitive Theory) | Using patient cases under feedback from an expert, the GPs will:  - practice their abilities to identify situations where a proactive intervention would be beneficial and how to do it  - work in pairs to train their ability to guide patients to self-care and the GPs’ skills in planning and completing follow-up meeting and adjusting patient processes  - train their ability to assess the patient’s functional capacity |
|  | ***Enactive mastery experiences***  Providing increasingly challenging tasks with feedback to serve as indicators of capability.  (Social Cognitive Theory) | GPs are provided increasingly complex patient cases and given feedback by an expert to:  - acquire relevant knowledge about the patient and find possible solutions  - practice engaging in dialogue with patients regarding SAW in difficult situations.  - create solutions adapted to the patient |
| Perceived susceptibility |  |  |
| GPs will recognize that   - Patients with MSD are at risk for having a recurrent or chronic condition and negative work-related consequences - Patients who rely strongly on the GP are at risk for becoming passive and unable to manage their work-related situation - Patients with low levels of self-management are at risk for negative consequences of MSD and decreased likelihood of SAW - Patient’s risk for negative consequences of MSD increases with their lack of knowledge about their SAW rights and responsibilities - Patients on full-time sickness absence are at risk of long-term sickness absence and work termination - The patient is in a difficult life situation and may have different expectations about SAW - The patient’s likelihood of SAW may decrease if the solution is not specific or tailored to the patient and the workplace - The patient’s likelihood of SAW may decrease if the solutions are not specifically planed and communicated with the patient and relevant stakeholders - Lack of collaboration with other stakeholders may increase the risk for uncoordinated guidance and solutions for the patient. - The worker conveys messages from other stakeholders, and this entails the risk for information loss and misunderstanding - The patient’s likelihood of receiving out-dated advice increase with time | ***Consciousness raising***  Providing information, feedback or confrontation about causes, consequences, and alternative for a problem or a problem behaviour  (Health Belief Model) | Provides the GPs with up-to-date knowledge about MSD and SAW, including real-life cases, statistics, and expert comments |
|  | ***Shifting perspective***  Encouraging taking the perspective of the other  (Theories of Stigma and Discrimination) | GPs are encouraged to read and discuss patient stories to improve the GPs’ understanding of:  - how patients experience the GPs role in MSD prevention and treatment and SAW  - how information can be lost when the patient passes on information from other actors and how a lack of knowledge about opportunities may have negative effects on the patient’s process  - how patients may experience MSD and why they may demand a sick note  - why the patient’s processes may need continuous adaptations and follow-up |
|  | ***Environmental re-evaluation***  Encouraging combining both cognitive and affective assessments of how the presence or absence of a personal behaviour affects one’s social environment  (Trans-Theoretical Model) | GPs are presented with patient cases to illustrate:  - how the lack of a concrete plan increases the risk of a negative process for the patient  - how a lack of cooperation between stakeholders increases the risk of misunderstandings and unintended solutions |
| Outcome expectations |  |  |
| The GP expects that:   - Early intervention will increase the patient’s likelihood of SAW and decrease the likelihood of long-term sickness absence - Initiating a dialogue with the patient about self-management will increase the likelihood of SAW - Performing assessments of functional capacity will increase the patient’s likelihood of SAW by focusing on what the patient can do, rather than what the patient cannot do - Discussing SAW options with the patient will increase the patient’s likelihood of getting an individually tailored solution - Discussing SAW with the patient will reduce the patient’s likelihood of negative, work-related consequences - Advising according to guidelines will help the patient SAW - Specific solutions will increase the likelihood of workplace implementation - Active collaboration between stakeholder will increase the patient’s likelihood of SAW - The patient’s likelihood of SAW may increase with follow up consultations and adjustment of plans - Staying updated decrease the likelihood of the GPs being frustrated about the process | ***Environmental re-evaluation***  Encouraging realization of the negative impact of the unhealthy behaviour and the positive impact of the healthful behaviour  (Trans-Theoretical Model) | GPs are encouraged to discuss and share experiences on:  - the impact of early interventions on the patient’s needs for sickness absence and work function  - expected outcomes of a dialogue with patients about self-management on patient behaviour  - how to enquire work-related factors in dialogue with the patient and how to identify resources and options for the patients  - how proper information may aid in making a better assessment of the patient’s situation and contribute to finding a better solution for the patient  - how dialogue about reduced sick leave can lower the negative work-related consequences for the employee and how no dialogue can lead to increased sick leave and more work-related consequences.  - the benefits of an active cooperation between the stakeholders |
|  | **Shifting perspective** Encouraging taking the perspective of the other.  (Theories of Stigma and Discrimination) | GPs read and discuss patient stories to facilitate an understanding of how concrete plans increases the likelihood that they are implemented at the workplace |

MSD: Musculoskeletal disorders; GP: General practitioner; SAW: stay-at-work
